# Estimating Skin Cancer Risk: Evaluating Mobile Computer-Adaptive Testing

**DOI:** 10.2196/jmir.4736

**Published:** 2016-01-22

**Authors:** Ngadiman Djaja, Monika Janda, Catherine M Olsen, David C Whiteman, Tsair-Wei Chien

**Affiliations:** ^1^ School of Public Health and Social Work Institute for Health and Biomedical Innovation Queensland University of Technology Brisbane Australia; ^2^ Institute for Health and Biomedical Innovation Institute for Health and Biomedical Innovation Queensland University of Technology Brisbane Australia; ^3^ National Health and Medical Research Council Centre for Research Excellence Sun and Health (CRESH) Brisbane, Australia Australia; ^4^ QIMR Berghofer Medical Research Institute Brisbane Australia; ^5^ Research Department Chi-Mei Medical Center Tainan Taiwan; ^6^ Department of Hospital and Health Care Administration Chia-Nan University of Pharmacy and Science Tainan Taiwan

**Keywords:** computer adaptive testing, skin cancer risk scale, non adaptive test, Rasch analysis, partial credit model

## Abstract

**Background:**

Response burden is a major detriment to questionnaire completion rates. Computer adaptive testing may offer advantages over non-adaptive testing, including reduction of numbers of items required for precise measurement.

**Objective:**

Our aim was to compare the efficiency of non-adaptive (NAT) and computer adaptive testing (CAT) facilitated by Partial Credit Model (PCM)-derived calibration to estimate skin cancer risk.

**Methods:**

We used a random sample from a population-based Australian cohort study of skin cancer risk (N=43,794). All 30 items of the skin cancer risk scale were calibrated with the Rasch PCM. A total of 1000 cases generated following a normal distribution (mean [SD] 0 [1]) were simulated using three Rasch models with three fixed-item (dichotomous, rating scale, and partial credit) scenarios, respectively. We calculated the comparative efficiency and precision of CAT and NAT (shortening of questionnaire length and the count difference number ratio less than 5% using independent t tests).

**Results:**

We found that use of CAT led to smaller person standard error of the estimated measure than NAT, with substantially higher efficiency but no loss of precision, reducing response burden by 48%, 66%, and 66% for dichotomous, Rating Scale Model, and PCM models, respectively.

**Conclusions:**

CAT-based administrations of the skin cancer risk scale could substantially reduce participant burden without compromising measurement precision. A mobile computer adaptive test was developed to help people efficiently assess their skin cancer risk.

## Introduction

In Australia, skin cancers account for approximately 80% of all newly diagnosed cancers [1]. There are three main types of skin cancer: (1) melanoma (the most dangerous form of skin cancer), (2) basal cell carcinoma (BCC), and (3) squamous cell carcinoma (SCC). BCC and SCC are often grouped together as nonmelanoma or keratinocyte skin cancers. Australia’s incidence of skin cancer is one of the highest in the world: two to three times the rates observed in Canada, the United States, and the United Kingdom [2], with age-standardized incidence rates for cutaneous melanoma at 65.3 × 10^-5^ and 1878 × 10^-5^ for keratinocyte cancer [1]. From a population of only 23 million, more than 434,000 people are treated for one or more nonmelanoma skin cancers in Australia each year [1].

Ultraviolet radiation exposure from sunlight is the major causal factor for skin cancer [2]. Personal behaviors to reduce excessive sunlight exposure are important modifiable factors for the prevention of skin cancers. The World Health Organization recommends several suitable behaviors such as appropriate use of sunscreens, staying in the shade, covering with sun protective clothing, giving up sunbathing, and abstaining from using sunbeds [3].

### Requirement for Model-Data-Fit Detection

In practice, we do not know the real skin cancer risk for a person. Thus, assuming a person has characteristic attributes that correlate highly with the underlying construct of skin cancer, risk can be assessed through questions (ie, questionnaire items); for example, phenotypic measures such as freckles, hair color, eye color, tendency to burn, or behavioral factors such as attitudes to tanning and use of sunbeds. Using the responses to these items, it should be possible to create a unidimensional (ie, addable) scale to measure these attributes and calculate an overall skin cancer risk score. Ideally, such a score would be precise and characterized by a small standard error (SE).

Statistical validity is the correlation between each person’s measures (or scores) on a questionnaire and those persons’ unobservable true status [4]. Such unobservable variables (eg, true score or behaviors relating to sun protection and sun exposure) are considered latent traits (ie, exists but cannot be directly observed). The question is how to obtain optimal correlation (or validity) between the items when the true score is unknown. Rasch models [5] can be a gateway to assess how well the items measure the underlying latent trait [6-8]. That is, a unidimensional scale can be verified by Rasch analysis: when the data fit to the Rasch model, all items can be added.

Questionnaires that are built and tested using the Rasch model have become common in educational assessment for many years but are now also increasingly appreciated in health assessment, including measures of patient outcomes (quality of life, pain, depression) and other diverse latent traits such as perceptions of patient hospitalization and nurse bullying [9,10]. We previously applied the Rasch model to the assessment of the quality of an instrument to measure attitudes to skin self-examination [11]. Rasch analysis allows researchers to calculate a precise estimate of the latent trait by assessment of unidimensionality of the items, assessment of differential item functioning [12] (eg, probability of giving a certain response on an item by people from different groups with the same latent trait), and the possibility of transferring static questionnaires to computer adaptive testing (CAT) [13].

### Multimedia Graphical Representations to Improve Patients’ Health Literacy

Patients’ health literacy is increasingly recognized as a critical factor affecting patient-physician communication and health outcomes [14], as a mediator for cancer screening behavior [15], and as a pathway between health literacy and cancer screening [16]. Adults with below basic or basic health literacy are less likely than adults with higher health literacy to get information about health issues from written sources (eg, newspapers, magazines, books, brochures, or the Internet) and more likely than adults with higher health literacy to get a lot of information about health issues from radio and television [17]. A mobile CAT with multimedia graphical representations (ie, similar to radio and television) could increase awareness of the risk of developing skin cancer (ie, health literacy) and motivate patient-physician communication and subsequently behavioral change. However, no mobile CAT app with graphical representations has been available until now.

### Study Aims

Using data from a large cohort study of skin cancer from Queensland, Australia [18], we conducted a simulation study with a methodological focus to apply Rasch models to an existing skin cancer risk questionnaire. Further, we sought to compare static (nonadaptive) presentation as commonly used in paper and pencil questionnaires versus computer adaptive testing (CAT) for its precision in measurement. We hypothesized that compared to nonadaptive testing (NAT), CAT would result in greater precision (lower SE) for a similar item number or a shorter questionnaire of similar SE.

## Methods

### Data Source

**Figure 1:**
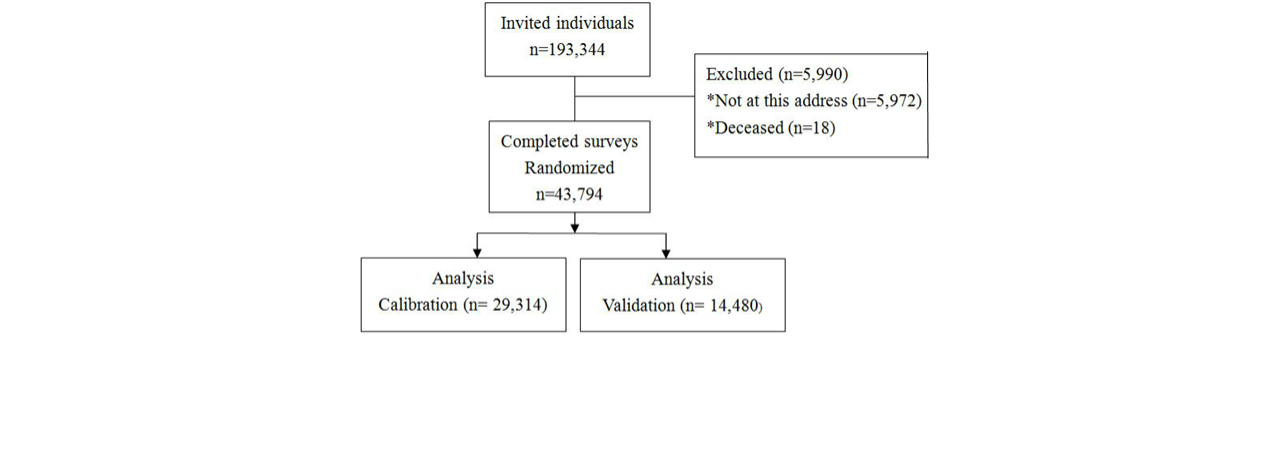
Sample selection flowchart.

**Figure 2:**
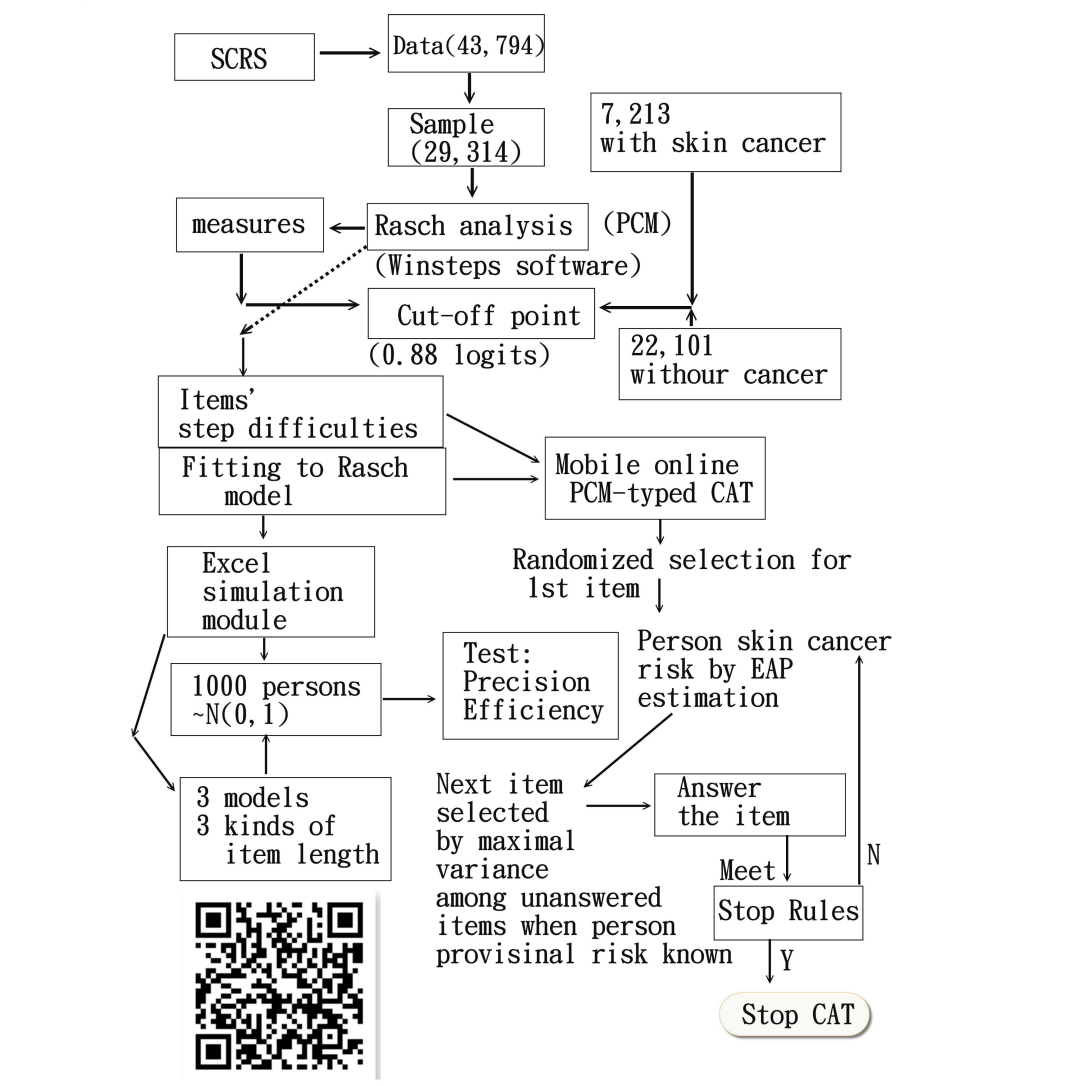
Study simulation and CAT flowchart (interested readers can run a test of the mobile CAT through the QR code).

De-identified data from the QSkin Sun and Health study baseline questionnaire were used [18]. This is a population-based cohort study of 43,794 men and women aged 40-69 years randomly sampled from the population of Queensland, Australia, in 2011 (Figure 1). We randomly partitioned the data into a calibration dataset (two-thirds, n=29,314) and a validation dataset (one-third, n=14,480). In the calibration dataset, 7213 participants had a history of skin cancer and 22,101 participants did not (Figure 2).

Approval for this study was obtained from the QIMR Berghofer Medical Research Institute Human Research Ethics Committee (approval #P1309). Participants joined the study by completing consent forms and the survey and returning them in a reply-paid envelope. Participants completed two consent forms. The first consent form covered the use of information provided in the survey, permission for data linkage to cancer registries, pathology laboratories, and public hospital databases. The second consent form gave permission for data linkage to Medicare Australia (Australia’s universal national health insurance scheme) to ascertain whether or not participants had developed skin cancer.

The baseline questionnaire consisted of 46 items and was answered by all QSkin participants. All items were examined using the Rasch Partial Credit Model (PCM) [19] (Figure 2). For optimal fit, the Rasch model requires a unidimensional measurement with criteria of Infit and Outfit mean square errors of each item ˂1.5 [20]. PCM allows for items to have a variable number of thresholds and step difficulties in contrast to the more commonly used Rating Scale Model (RSM) [8,9,21], which requires all items to use the same response categories.

For item invariance, the item estimation should be independent of the subgroups of individuals completing the questions and should work equally across populations [22]. Items not demonstrating invariance are commonly referred to as exhibiting differential item functioning (DIF) [23,24] or item bias. The chi-square test used for detecting DIF was computed from a comparison of the observed overall performance of each trait group on the item with its expected performance [25]. Its probability (eg, *P*<.05) reports the statistical probability of observing a chi-square value when the data fit the Rasch model. We used WINSTEPS [26] to detect items above the thresholds for DIF.

In addition, the category structure for each of the items in the skin cancer item bank should display monotonically increasing thresholds following the Linacre’s guidelines [27] to improve the utility of the resulting measures.

### Determining a Cut-Off Point of Skin Cancer Risk

**Figure 3:**
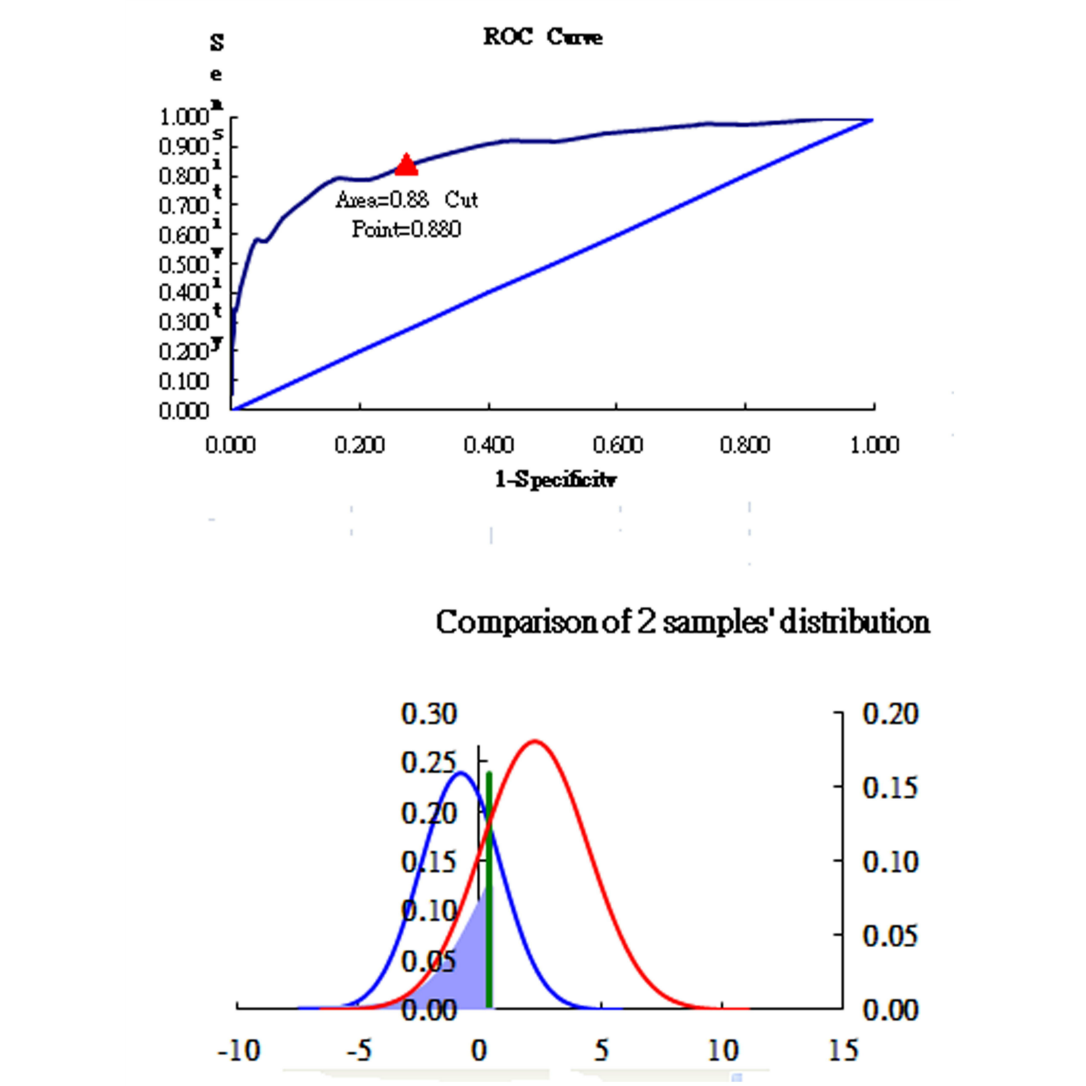
Cut-off point determined.

Traditionally in clinical practice, researchers use C-statistics, or area under the receiver operating characteristic (ROC) curve to plot the true positive rate (sensitivity) against the false positive rate (1 - specificity) at various threshold settings [28]. In this study, we plotted two sample normal distributions incorporated with ROC in Figure 3 when their means and standard deviations were known.

Much information such as cut point, area under ROC curve, and a graphical vertical bar showing cut points can be displayed on a plot. WINSTEPS software [26] was used to estimate means and standard deviations of cases with and without previous skin cancers to determine a cut-off point of skin cancer risk with maximal sensitivity and specificity in MS Excel (Figure 3). Providing the cut-off points in graphical form makes the results clear and easily understandable for readers or clinicians to interpret.

### Mobile Computer Adaptive Testing Designed for Examining Personal Skin Cancer Risk

The CAT item bank (fitting to Rasch model’s requirement regarding unidimensionality, local dependence, and monotonicity as well as DIF absence on gender) was constructed, consisting of all 31-item parameters obtained from the calibration using WINSTEPS [26].

To start the CAT, an initial item was selected randomly from the item bank. Using this initial item, a provisional person measure was estimated by the expected a posteriori (EAP) method [29] in an iterative Newton-Raphson procedure [9,30]. After each item was answered, EAP was recalculated, until the final score for the person was determined by the maximum of the log-likelihood function before terminating the CAT (Figure 2). The next item selection was based on the highest Fisher information (ie, item variance) of the remaining unanswered items interacting with the provisional person measure.

Two termination rules were set. The first was a minimum standard error of measurement (SEM) of 0.47 required for stopping the CAT. This SEM was set based on the internal consistency of the calibration sample (Cronbach alpha=.78). SE_i_ was the person SE of the estimated measure according to their item variances of the finished items on CAT, where SEM=SD × sqrt (1 - reliability) and SE_i_=1/sqrt(Σinformation[i]), where *i* refers to the CAT finished items responded to by a person [31], and SD is the person standard deviation of the derivation sample of 29,314 cases. The second termination rule was that each person must answer at least 10 items according to a simulation study on the data bank for attaining a minimal average personal reliability at a desired level (eg, 0.78) [32].

### Simulation to Compare Efficiency and Precision of Computer Adaptive Testing and Nonadaptive Testing

Using the item parameters generated from the derivation cohort, 1000 cases following a normal distribution (mean logit 0, SD logit 1) were simulated [33-35] using three Rasch models (ie, dichotomous, 5-point RSM, and PCM) with three respective fixed-item scenarios (ie, 10, 20, and 30 items; see Tables 1-3).

**Table 1:** 10, 20, or 30 items in static NAT format.

Datasets	Dichotomous		RSM		PCM	
	Mean	SE	Mean	SE	Mean	SE
10 items	-0.007	0.829	0.03	0.414	-0.179	0.398
20 items	-0.008	0.555	0.02	0.289	-0.19	0.272
30 items	0.045	0.439	-0.039	0.235	-0.084	0.224
CAT	-0.021	0.613	0.021	0.361	-0.154	0.32

**Table 2:** Precision of CAT.

Precision	Dichotomous		RSM		PCM	
	Diff. (%)^a^	Corr.^b^	Diff. (%)^a^	Corr.^b^	Diff. (%)^a^	Corr.^b^
10 items	0.40	0.863	0.30	0.952	0.00	0.931
20 items	0.00	0.957	0.00	0.988	0.00	0.986
CAT	0.13	0.925	0.05	0.958	0.10	0.946

^a^Diff. (%): Different number ratio compared to the 30-item dataset.

^b^Corr: Correlation coefficient of person theta to NAT.

**Table 3:** Efficiency of CAT.

Efficiency	Dichotomous		RSM		PCM	
	CAT item length	%^a^	CAT item length	%^a^	CAT item length	%^a^
CAT	15.55	48.20	10	66.70	10.13	67.32

^a^Efficiency=1 - CIL/30.

To allow testing of dichotomous and 5-point rating scale Rasch models, all item (or step) difficulties were converted from the calibrated results of the PCM. The overall difficulty for each item was designated to be the respective threshold of the dichotomous scale. In contrast, the step difficulties of the 5-point RSM [21] ranged from -2 to 2, with an advance 1.0 logit interval added to the overall difficulty of the respective item as to the PCM.

We calculated the comparative efficiency and precision for CAT and NAT by varying the number of items presented (10, 20, and 30 items) and by testing the difference in precision and efficiency compared to answering all available 31-items using independent *t* tests to count different number ratio less than 5% as shown in the following formula [36], respectively:

t=|θ_cat_ - θ_30_|/sqrt(SE^2^_cat_+ SE^2^_30_)

In addition, a comparison of average person SEs achieved across all different conditions was made to verify precision for CAT and NAT. We ran an author-created Visual Basic for Applications module in MS Excel to conduct the simulation study (Figure 2) and mobile CAT.

## Results

### Determining a Cut-Off Point

The mean and SD of skin cancer risk for participants without skin cancer (mean -0.79, SE 1.67) or with skin cancer (mean 2.29, SE 2.21) were calculated and used to determine the optimal cut-off point at 0.88 logit with sensitivity at 0.79 and specificity at 0.74. Using this cut-off, the area under the ROC curve was 0.88 (see Figure 3).

### Simulation to Compare Efficiency and Precision of Computer Adaptive Testing and Nonadaptive Testing

**Figure 4:**
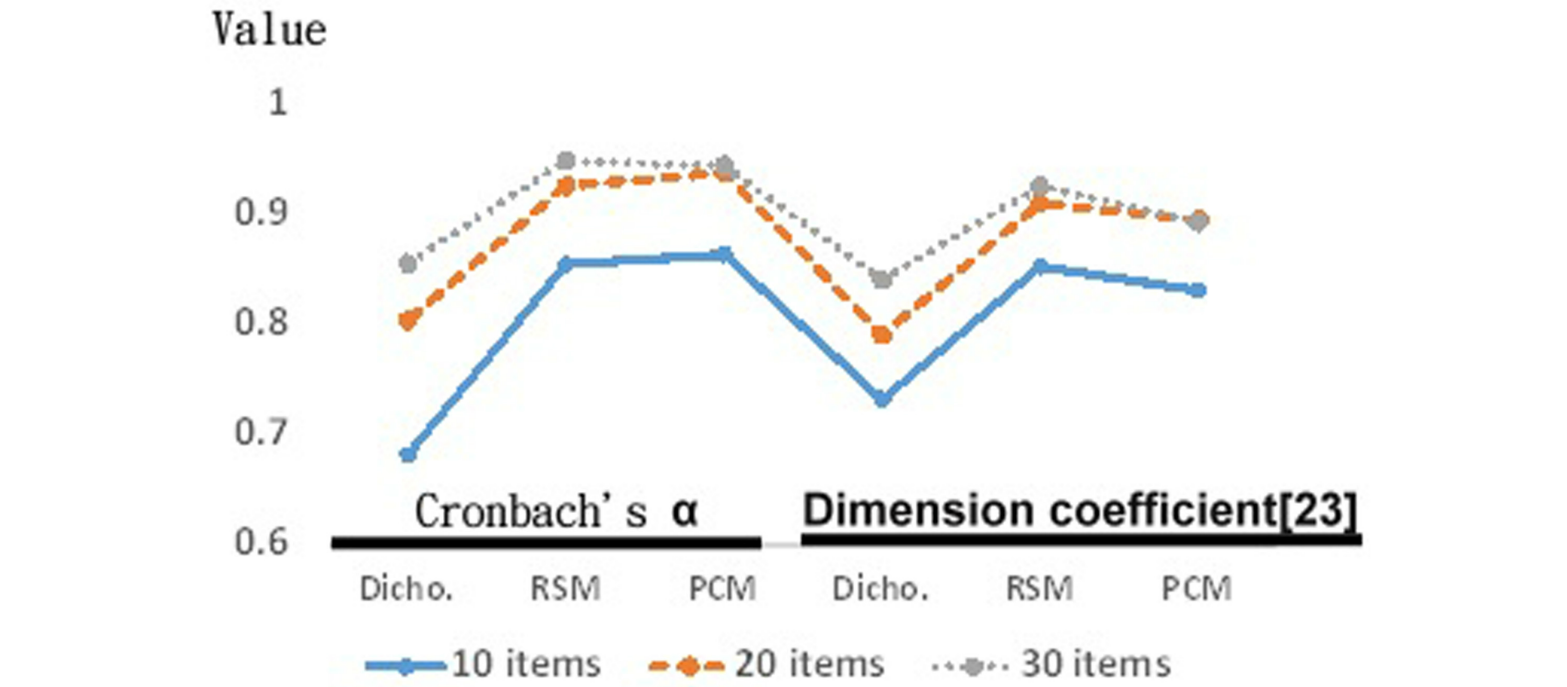
Simulation data generated with 3 Rasch models.

Using simulation data, we found that using more items yielded higher Cronbach alpha scores (Figure 4). Dichotomous scales had the lowest Cronbach alpha and dimension coefficient [37]. The PCM scales had the highest Cronbach alpha. The RSM scales gained the highest dimension coefficient.

**Figure 5:**
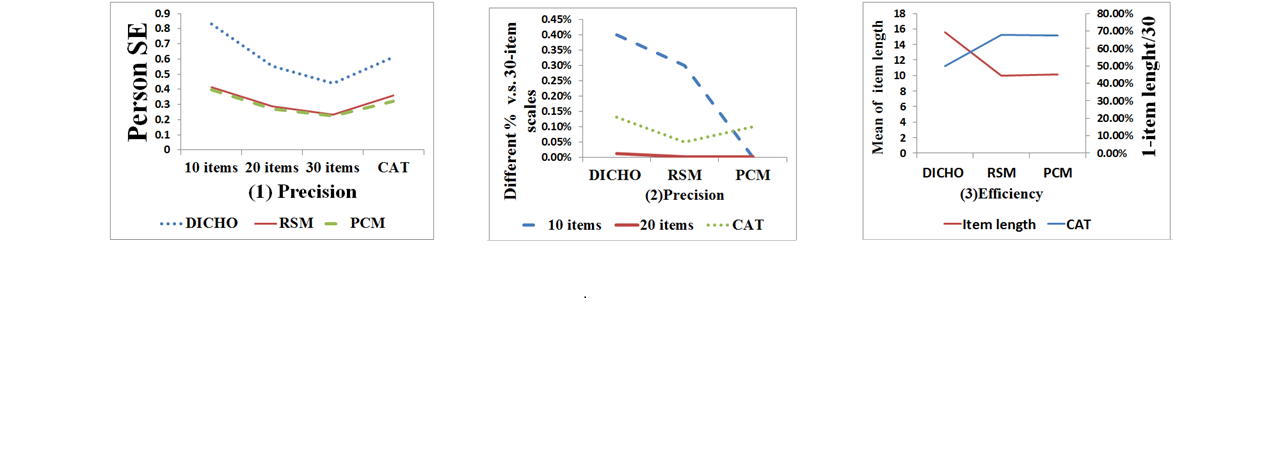
Efficiency and precision of CAT, compared to using 10, 20, or 30 items in static NAT format.

As shown in Figure 4, CAT gained a relatively smaller SE corresponding to item length (ie, compared to NAT, shorter CATs result in larger SE). At equivalent precision, CAT reduces the response burden by 48.20%, 66.70%, and 66.20%, respectively for dichotomous, RSM, and PCM models (Figure 5).

### Mobile Computer Adaptive Testing Evaluating Skin Cancer Risk

**Figure 6:**
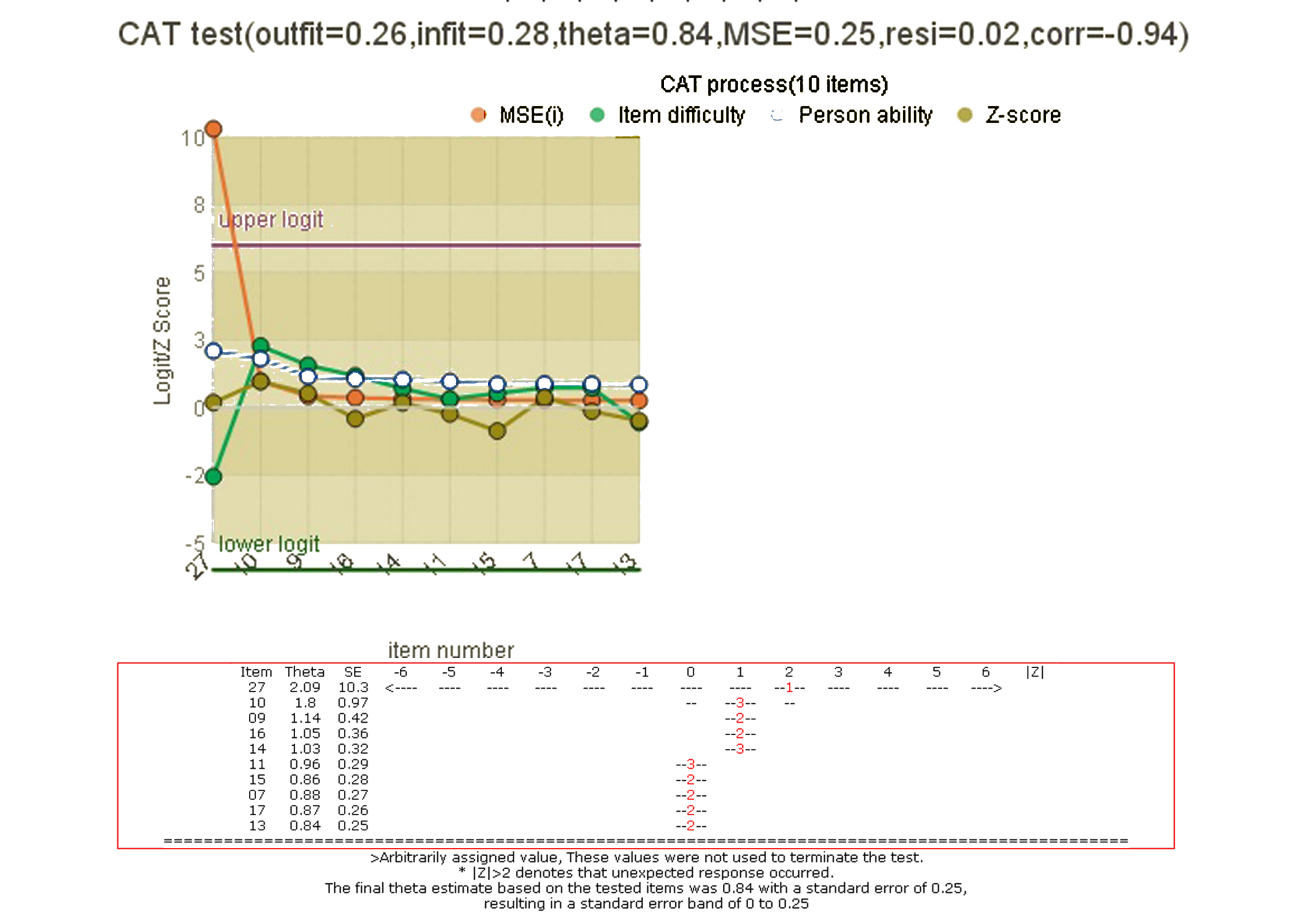
A graphical CAT report shown after each response (top) and the more item length, the less standard errors in CAT process (bottom).

We developed a mobile CAT survey procedure (see QR code in Figure 2 and Multimedia Appendix 1) to practically demonstrate the newly designed PCM-type CAT app in action. The CAT process was demonstrated item by item and is shown at the top of Figure 6. Person theta is the provisional ability estimated by the CAT module. The mean square error at the bottom of Figure 6 was generated by the formula of 1/sqrt(Σinformation[i]), where *i* refers to the CAT presented items responded to by a person [31]. In addition, the residual at the top of Figure 6 was the average of the last five change differences between the pre-and-post estimated abilities on each CAT step. CAT will stop if residual value ˂0.05. The “corr” refers to the correlation coefficient between the CAT estimated measures and the step series numbers using the last 5 estimated theta values. The flatter of the theta trends means the higher probability of the person measure convergent to a final estimation.

## Discussion

### Principal Findings

We used two different approaches to measure risk of skin cancer: nonadaptive testing and computer adaptive testing. Using data from a very large cohort of more than 43,000 people, we were able to show that our scale was able to accurately identify people at highest risk for skin cancer. On our risk scale, we identified a very high discriminatory accuracy of 0.88 (ie, the proportion of area under ROC curve) using a cut-off of 0.88 logits (the higher, the worse). Using CAT results in a smaller SE at high efficiency (fewer items answered), and therefore without compromising test precision, reduces response burden by 48.20%, 66.70%, and 66.20% for dichotomous, RSM, and PCM models, respectively. A prototype mobile online CAT for evaluating skin cancer risk has been developed and could be used to assess skin cancer risk at considerable reduction of respondent burden.

Consistent with the literature [8,9,30,34,35], the efficiency of CAT over NAT was supported for this skin cancer risk scale. We confirm the PCM-type CAT (ie, different from others by using simpler Rasch family models) requires significantly fewer items to measure a person’s risk than NAT but does not compromise the precision of measurement. This mobile assessment could be used to quickly estimate a person’s skin cancer risk and educate them about the need for skin protection on a personal level [38-40]. We confirm that participants with a history of skin cancer had a higher mean score of responses than those without a history of skin cancer.

### Implications

Patients’ health literacy (eg, understanding their own skin cancer risk) is increasingly recognized as a critical factor affecting patient-physician communication and health outcomes [14]. Adults with below basic or basic health literacy are more likely than adults with higher health literacy to get information about health issues from multimedia graphical representation [17], rather than the traditional newspapers, magazines, books, brochures, or *pamphlets*. A brief CAT such as the one we developed could be used to inform people quickly about their skin cancer risk and how to improve their sun protection behaviors.

This CAT module is a practical tool that can gather responses from patients efficiently and precisely. The tool offers diagnostics that can help practitioners assess whether responses are distorted or abnormal. For example, outfit mean-square values of 2.0 or greater suggest an unusual response. In instances where responses do not fit with the model’s requirement, they can be highlighted for suspected cheating, careless responding, lucky guessing, creative responding, or random responding [41]; otherwise, one can take follow-up action [8,34,35] if the result shows a high cancer risk. For example, if a person’s measure/risk is 1.0 logit (ie, log odds), their probability of developing skin cancer approaches 0.53(=exp(1-0.88)/(1+exp(1-0.88)). Interested readers can run a test of the mobile CAT through the QR code shown in Figure 2.

A mobile online CAT could be used for evaluating skin cancer risk and might reduce the item length in clinical settings. The CAT can be improved in the future by expanding the item pool allowing use among more diverse samples. It must be noted that (1) item overall (ie, on average) and step (threshold) difficulties of the questionnaire must be calibrated in advance using Rasch analysis or other item response theory models before creating an item bank, (2) pictures used for the subject or response categories for each question should be well prepared with a Web link that can be shown simultaneously with the item appearing in the animation module of CAT, and (3) the model can be used for many kinds of models based on item response theory.

### Strengths and Limitations

There are two major forms of standardized assessments in clinical settings [42]: (1) a traditional self-administered questionnaire, and (2) a rapid short-form scale [43,44]. Each has its advantages and drawbacks. Traditional pencil-and-paper questionnaires have a large respondent burden, often because they require patients to answer questions that do not provide additional information about their risk of disease in order to achieve adequate precision measurement [45]. CAT can target the optimal question for a specific person and therefore end at an appropriate number of items more economically according to the required SE (or say, criterion of person reliability). However, along with the advantages offered by CAT, there are some drawbacks as well, such as impossibility of estimating the ability in case of all extreme responses, CAT algorithms requiring serious item calibration, several items from the item bank being overexposed, and other test items not being used at all [46].

The strengths of this study include its very large sample size of more than 40,000 participants, permitting detailed analysis of the performance of questionnaire items and the ability to further test the performance of the items in a validation dataset. We simulated data by varying the types of models and item length to execute the CAT. (Interested readers who wish to see the video demonstration or use the MS Excel-type module can contact the corresponding author).

As with all forms of Web-based technology, advances in mobile health (mHealth) and health communication technology are rapidly emerging [47]. Use of mobile online CAT is promising and worth considering in many fields of health assessment, similar to its prominent role in education and staff selection testing. However, several issues should be considered more thoroughly in further studies. The scale’s Cronbach alpha (=.78 yielded by studied 29,314 cases), sensitivity at 0.79, and specificity at 0.74 are slightly low. Second, the CAT module has a potential limitation for people using languages other than English because the interface may need to be modified for use in real world. A multiple language interface should be developed in the future. Third, the CAT graphical representation shown in Figure 6 might be confusing and difficult to interpret for people unfamiliar with CAT and may need to be improved to become a standard part of CAT routine.

### Conclusions

The PCM-type CAT for skin cancer risk can reduce respondents’ burden without compromising measurement precision and increases endorsement efficiency. The CAT module can be used for mobile phones and easy online assessment of patients’ disease risks. This is a novel and promising way to capture information about skin cancer risk, for example while waiting outside physician consultation offices.

## Appendix

Multimedia Appendix 1 Demonstration of an online CAT.

## Abbreviations

BCC: basal cell carcinoma
CAT: computer adaptive testing
DIF: differential item functioning
NAT: nonadaptive testing
PCM: Partial Credit Model
ROC: receiver operating characteristic
RSM: Rating Scale Model
SCC: squamous cell carcinoma
SE: standard error
SEM: standard error of measurement
